# An Unrecognized Fracture of the Neck of the Femur

**Published:** 1879-11

**Authors:** C. C. Wyckoff

**Affiliations:** Buffalo


### "AN UNRECOGNIZED FRACTURE OF THE NECK OF THE FEMUR."

*To the Editors of the Buffalo Medical and Surgical Journal:*—Will you oblige me by publishing the accompanying abstract from the Hospital records; which is a part of the history (omitted) of the case published in the October number of your valuable Journal, as "An Unrecognized Fracture of the Neck of the Femur." Truly yours, C. C. WYCKOFF, M. D.

BUFFALO, Oct. 16, 1879.

### CASE 7,534.

KATIE SPEIGLE, age 18 years, American, single, domestic. Entered the hospital Jan. 16, 1878; had been sick 19 days; taken at night with acute pain in the shoulder; in the morning it had changed to inner side of thigh, well up; part was inflamed and very painful; her physician "suspected spinal fever," or rather the premonitions of such; later it was that dead bone was the active agent, and an abscess anticipated; at the time of entering the hospital, thigh was swelled and sensative, the knee well flexed and the whole limb remarkedly everted and fixed in position. This she said was the most comfortable position she could assume, and she claimed to have taken this position from the first. To move the limb caused great pain in the thigh. Investigation proved that she had an abscess in the thigh when five years old, which her parents attributed to measles; but it is learned that subsequently to the measles and previously to the trouble in the thigh, the patient had a fall on the pavement, and was immediately afterwards confined to her bed by the "abscess." This was the only illness she had experienced. After mature consideration it was decided that the patient was suffering from rheumatism, and she was given salicylic acid gr. x every 2 hrs. for 48 hrs., and then gr. x every 4

hrs. for several days, with anodynes. Pulse at entrance and afterwards was Jan. 16 to 29th, A. M., 94, 114, 100, 102, 120, 100, 98, 100; P. M., 108, 90, 90, 91, 108, 110, 116, 100. Temp. ranged from 99½° to 101½°. By the use of acid salicylic inflammation subsided at once. Rochelle salts were given also. In a few days swelling appeared in the knee joint, which also pitted and presented all the signs of rheumatism.

Jan. 30th. Vini colchici sem. gtt. xv., every 4 hours given in place of the salicylic acid. Rheumatism gradually subsiding.

March 1st. Rheumatism well but foot greatly everted, and measurement showed the leg two inches short, and while upon the sound side the distance from great trochanter to ant. sup. spin. process was 4½ in., upon this side it was 6½ in.

March 10th. Extension by weights and pully 12 lbs. continued 13 days. Extension relieved her of pain, previously felt in hip-joint, for a few weeks only, and it was thought the foot took a more natural position.

March 23d. Leg manipulated by Dr. Gay, which resulted in lessening the shortening 1 in., and lessening distance from trochanter to spine from 6½ to 4½ or 5 in. Foot was also brought into natural position and readily retained there. Extension was again applied, 8 lbs. wt. being used. Before manipulation she was partly etherized but took the anesthetic very badly, so that insensibility to pain could not be established perfectly with proper regard to safety. She was removed to a hard bed and given morphia sulph. gr. ¼. Pulse from March 23d and afterwards was, A. M., 160, 130, 108, 120, 118, 120; P. M., 158, 130, 108, 120, 118, 120. Temp. A. M. and P. M., 102½°—102°, 101°—101½°—99°. Some dyspnœa and pain in chest and slight hemoptysis occurred the succeeding evening; digitalis given; gradually improved; leg all right save pain in hip-joint; morphine given.

April 1. Leg one inch short. The patient says her father had told her that this leg was always shorter since the "abscess." Her mother did not know of it, and no positive proof was given.

